# A New Perspective on Nasal Microbiota Dysbiosis-Mediated Allergic Rhinitis: From the Mechanism of Immune Microenvironment Remodeling to Microbiota-Targeted Therapeutic Strategies

**DOI:** 10.3390/ijms262412061

**Published:** 2025-12-15

**Authors:** Lijun Du, Xiangning Cheng, Bo Liu, Yuzhe Hao, Ziyi Long, Qianxue Hu, Bingyue Huo, Tianjian Xie, Qing Cheng, Yue Zhou, Jianjun Chen

**Affiliations:** Department of Otorhinolaryngology, Union Hospital, Tongji Medical College, Huazhong University of Science and Technology, Wuhan 430022, China; 18872911148@163.com (L.D.); m202476362@hust.edu.cn (X.C.); liu117378@163.com (B.L.); d202482307@hust.edu.cn (Y.H.); lzy000414@163.com (Z.L.); 15007228650@163.com (Q.H.); u201810289@hust.edu.cn (B.H.); tianjian_xie@hust.edu.cn (T.X.); cqjj74@163.com (Q.C.)

**Keywords:** allergic rhinitis, nasal microbiome, potential mechanisms, therapeutic strategies

## Abstract

Allergic rhinitis (AR) is a common heterogeneous chronic disease characterized by high prevalence, complex pathogenesis, and susceptibility to multiple contributing factors. Currently, its prevalence ranges from 20% to 30% in adults and reaches up to 40% in children. Extensive research has confirmed significant differences in nasal microbiota composition between AR patients and healthy individuals, most notably alterations in the abundance of four dominant phyla: *Actinobacteria*, *Bacteroidetes*, *Firmicutes*, and *Proteobacteria*. Among these, the most striking abundance alterations occur in *Staphylococcus aureus* and *Streptococcus salivarius* within the nasal mucosa of AR patients, suggesting a critical role of nasal microbiota in AR initiation and progression. In response, researchers have proposed microbiome-targeted therapeutic strategies. For example, nasal local administration of probiotics (e.g., *Lactobacillus* and *Bifidobacterium*) aims to reshape the nasal microbiota. Additionally, protective bacteria such as *Corynebacterium accolens* and *Dolosigranulum pigrum* can inhibit pathogenic bacteria, thereby correcting microbial dysbiosis and alleviating AR symptoms. This review summarizes the composition of the nasal microbiota, the latest research progress on its association with AR, and the underlying potential mechanisms. It provides novel insights and potential therapeutic strategies for the prevention and treatment of AR.

## 1. Introduction

Allergic rhinitis (AR) represents the most prevalent type of rhinitis, differing from other subtypes in its inherent allergic etiology and pathological response [1,2]. AR prevalence has risen steadily over the past several decades; epidemiological investigations indicate it now impacts 20% to 30% of adults and as many as 40% of children [2,3]. In Poland, Breborowicz et al. reported that 16.7% of children aged 6–7 years suffer from AR, while Emeryk et al. observed that 3.6% of children aged 8–15 years have perennial AR and 6.2% have seasonal AR [4]. Additionally, a survey on AR prevalence in Yinchuan, China, found a prevalence of 17.1% (202/1183) among middle-aged individuals (40–59 years) and 19.0% (314/1653, χ^2^ = 10.36, *p* = 0.023) among young adults (18–39 years) [5]. Thus, there are differences in the prevalence rates of AR between children and adults, which may result from the combined effects of age, environment, and immune mechanisms. AR is also a costly condition requiring long-term control. Its disease burden encompasses the severity of nasal symptoms, compromised quality of life across multiple domains, and a range of comorbid conditions [3,4]. Within the European Union, the annual economic burden from absenteeism and presenteeism due to untreated or inadequately managed AR is projected to be between 55 and 151 million Euros [5]. AR is a common disease associated with IgE-mediated type 1 hypersensitivity and type 2 immune-mediated inflammation [6]. Once viewed as a condition confined to the nasal cavity, AR is now understood to be a manifestation of systemic airway dysfunction, often presenting with asthma as a comorbidity [7]. Notably, the pathological mechanisms and treatment of AR remain incompletely understood and require further investigation.

Human microbiota is a large and complex microecosystem composed of trillions of microorganisms, including bacteria, archaea, viruses, and fungi [8]. Among these, bacteria have been the primary focus of research due to their high abundance, easy detectability, and cultivability. With the development of high-throughput sequencing technologies, metagenomics has become an important method that not only predicts the abundance and richness of mucosal microbial communities but also reveals their roles in health and disease [9]. Studies on the interaction between the mucosal microbiome and the host are also increasingly emphasizing the contribution of specific microbiotas to mucosal immune responses [10]. The nasal microbiota denotes the microbial community that colonizes nasal cavities, including the anterior nares, middle nasal meatus, and inferior nasal concha mucosa [11]. The paranasal sinus microbiota represents the microbial assemblage inhabiting paranasal sinus cavities (i.e., maxillary, frontal, ethmoid, sphenoid sinuses) and associated mucosal surfaces [12]. Both are integral components of the upper respiratory tract microbial ecosystem, with bidirectional microbial migration and communication. Consequently, the paranasal sinus microbiota is included when addressing the nasal microbiota in this research. A diverse and balanced microbial community within the nasal cavity and paranasal sinuses is critical for maintaining immune function and mucosal barrier integrity. Disruption of this balance (i.e., dysbiosis) has been linked to the onset of various disorders, including allergic rhinitis (AR), chronic rhinosinusitis (CRS), and nasal malignant tumors. Moreover, eosinophilic inflammation, Th17 gene expression, neutrophilic inflammation, and allergic inflammation markers are all associated with differences in airway microbiota composition—highlighting the link between the nasal microbiome and nasal tract immune disorders (including AR) [13,14]. Multiple observational studies have found that the nasal microbiota of adults is dysregulated during airway allergic inflammation, with certain microbial taxa becoming dominant [15,16]. In contrast to adults, microbiome research related to AR has been relatively understudied in children and adolescents [17]. Studying the spectrum of early microbiome changes may open new ways to prevent and predict disease in the future. However, current research on the nasal microbiome and allergic rhinitis (AR) remains relatively limited; for instance, the mechanisms underlying the differences in nasal microbiome characteristics have not yet been clarified.

Regarding the pathogenesis of allergic rhinitis (AR) involving the nasal tract microbiota, most studies have indicated that it is primarily manifested as microbial dysbiosis-driven disruption of the local mucosal barrier and systemic immune imbalance. Specifically, structural alterations in specific microbial communities can directly affect the integrity of the nasal epithelial mucosa and activate innate immunity; meanwhile, microbial metabolites mediate functional reprogramming of distal immune organs via the circulatory system [13,18,19]. Therefore, this review will systematically sort out the core compositional features of the nasal microbiome, focus on summarizing the latest research findings on its association with AR, and conduct an in-depth analysis of the key mechanisms underlying the nasal microbiome’s regulation of AR. Among these mechanisms, core bacteria represented by *Staphylococcus aureus* and *Streptococcus salivarius* play a critical role by regulating local inflammatory responses and disrupting immune homeostasis. Meanwhile, viruses such as rhinoviruses and fungi such as *Alternaria alternata* can also jointly participate in the pathogenesis of AR by impairing the integrity of the nasal mucosal barrier and activating abnormal immune responses. Additionally, this review will elaborate on the potential of microbial modulation as a therapeutic strategy.

## 2. Methods

To systematically review the associations between nasal mucosal microbiota (including paranasal sinus microbiota) and allergic rhinitis (AR), the molecular mechanisms by which microbiota regulates the pathogenesis of AR, and the potential value of microbe-targeted interventions (e.g., probiotics), this study conducted a systematic literature search. Included study types were observational studies (cross-sectional, cohort, or case–control studies) and experimental studies (e.g., animal model studies of AR, randomized controlled trials [RCTs] of probiotic interventions). Exclusion criteria were applied to minimize confounding effects from other comorbidities: studies focusing on atopic dermatitis, food allergies, or intestinal diseases; and studies focusing solely on respiratory microbiota unrelated to the nasal/paranasal sinus mucosa (e.g., oropharyngeal or tracheal microbiota). Relevant literature published up to September 2025 was searched in databases, including PubMed, Web of Science, and Google Scholar. Studies involving analysis of nasal microbial composition, the association between nasal microbiota and AR pathogenesis, or probiotic-targeted therapies for AR were screened, summarized, and systematically analyzed.

## 3. The Composition of Nasal Tract Microorganisms

The human body is not a sterile environment. Instead, it harbors a vast and diverse assemblage of symbiotic microbes. These microbes colonize numerous body cavities and surface regions, such as the respiratory tract, digestive tract, urinary tract, female reproductive system, skin, and nasal mucosal surfaces. The mucosal surfaces of the nasal tract are physiologically colonized by their own collection of microbes, the microbiota, including pathogenic, commensal, and symbiotic microorganisms [19,20,21,22,23,24,25]. The nasal tract microbiome includes not only a variety of microorganisms but also their genetic material and metabolites, such as proteins, lipids, and carbohydrates.

Studies have shown that a balanced nasal microbiota plays a crucial role in reducing the incidence of allergic rhinitis (AR). Although defining a “balanced microbiota” remains challenging, it is generally regarded as the counterpart of dysbiosis [26]. This imbalance in the composition and metabolic activity of the body’s microbiota can manifest in multiple ways, such as the loss of beneficial microorganisms or the overgrowth of potential pathogens [27]. Identifying beneficial microorganisms and potential pathogens is not straightforward. The nasal cavity of healthy individuals typically harbors a rich array of bacterial genera in the absence of overt symptoms. Many studies report that the nasal microbiome of healthy humans is primarily composed of the phyla *Actinobacteria*, *Bacteroidetes*, *Firmicutes*, and *Proteobacteria*, with representatives of the genera *Bifidobacterium*, *Corynebacterium*, *Staphylococcus*, *Streptococcus*, *Dolosigranulum*, and *Moraxella predominating* [19,20,21,22,23]. It can thus be inferred that these taxa contain potentially beneficial bacterial strains. Since the nasal cavity is continuously exposed to the external environment [20], and its mucosa is lined with diverse types of epithelial cells, it creates a heterogeneous microenvironment for the microbiota. In addition, host genetic factors regulate this process, ultimately resulting in highly complex microbial diversity [28,29,30]. Knowledge of the nasal mucosal microbiome in the pediatric population is very limited. Despite the limited number of studies, several bacterial taxa have been identified as resident microbiota of the nasal mucosa in children and adolescents, such as *Acinetobacter*, *Corynebacterium*, *Dolosigranulum*, *Haemophilus*, *Moraxella*, *Staphylococcus*, and *Streptococcus* [31,32]. Furthermore, the development of the nasal microbiome is a complex multi-stage process. The microbiome exhibits variations across different anatomical sites (e.g., nasal cavity, nasopharynx, oropharynx) and undergoes continuous changes during the successional process from infancy to adulthood. Specifically, the composition of the microbiota may alter throughout an individual’s life cycle [24,33,34,35,36] (Table 1).

## 4. Characteristics of Nasal Tract Microbial Dysbiosis in Allergic Rhinitis

### 4.1. Characteristics of the Nasal Tract Microbiome in Pediatric Allergic Rhinitis

There are significant differences in the nasal bacterial communities between children and adults. Culture-based studies have confirmed that a variety of potential pathogenic bacteria can be frequently isolated from the nasopharynx of healthy children, including *Streptococcus*, *Haemophilus influenzae*, *Neisseria*, *Staphylococcus aureus*, *Actinomyces*, *Porphyromonas*, *Bacteroides*, *Prevotella*, *Peptostreptococcus*, and *Fusobacterium* [39]. In contrast, the dominant nasal microbiota in healthy adults are *Corynebacterium* and *Staphylococcus epidermidis* [40]. Such disparities may stem from the immature physiological structure of the nasal cavity and underdeveloped immune function in children, which render their microbial composition more susceptible to external environmental factors. Although current research on the nasal mucosal microbiota in children with allergic rhinitis (AR) remains relatively limited [31,32,41,42] existing evidence indicates that nasal microbiota dysbiosis in children with AR may be associated with increased sensitization to allergic diseases [42,43]. Furthermore, this dysbiosis is primarily manifested in two aspects: alterations in microbial diversity and abnormal abundance of specific bacterial genera.

The microbial diversity in children with allergic rhinitis (AR) is significantly reduced. Multiple studies have observed that the nasal microbial diversity of children/infants with AR is notably lower than that of healthy children. Morin et al. found that the bacterial diversity in the lower pharynx of AR-affected children at 1 month and 3 months of age was decreased, with a reduction in the abundance of beneficial bacteria such as *Lactobacillus* and *Bifidobacterium*. However, this study has certain limitations: the samples were only derived from children of European ancestry, and the ethnic generalizability of the results remains to be verified [18,44]. After comparing with the healthy control group, Ta et al. pointed out that in infants who developed AR within the first 18 months of life, the nasal abundance of commensal bacteria (e.g., *Veillonella* and *Roseburia*) was reduced, and the overall microbial diversity indices (Shannon index and Simpson index) were 20–30% lower than those of healthy infants [44]. Furthermore, Chiu et al. further discovered that the significant decrease in nasal microbial diversity in children with AR was associated with their susceptibility to house dust mites (HDM). In this study, the abundance of *Moraxella* was significantly increased (1.8-fold higher than that in the healthy group), and this bacterium has been confirmed to promote the secretion of inflammatory cytokines (IL-4 and IL-13) via activation of the TLR4 signaling pathway—thereby participating in the interaction between HDM sensitization and rhinitis [42]. The above evidence suggests that resident commensal bacteria may behave as pathogenic agents when exposed to environmental changes (e.g., allergen sensitization), which may disrupt the nasal microbiome and exacerbate disease progression [45,46]. Notably, in the nasal cavity of healthy children, *Moraxella* and *Streptococcus* (predominantly *Streptococcus mitis*) are typically the dominant bacterial genera [47]. In contrast, this dominant community structure is disrupted in the nasal cavity of children with AR, with a significant increase in the abundance of *Staphylococcus aureus.* A case–control study involving 62 children with AR (case group) and 51 healthy children (control group) further verified the unique characteristics of the nasal microbiota in children with AR. By using 16S rRNA sequencing combined with FEAST (Fast Expectation-Maximization for Microbial Source Tracking) technology, the researchers identified 10 bacterial taxa with differential enrichment between the case and control groups (linear discriminant analysis [LDA] score > 2, *p* < 0.05). Among these, *Staphylococcus aureus*, *Staphylococcus epidermidis*, *Staphylococcus saprophyticus*, and *Staphylococcus haemolyticus* in nasal samples were the four bacterial species exclusively detected in children with AR. Conversely, among the six bacterial taxa with significantly higher abundance in the nasal cavity of healthy children, *Aggregatibacter aphrophilus*, *Cutibacterium acnes*, and *Moraxella* ranked among the top three. These findings indicate a significant association between the nasal microbiota and AR [40].

Moreover, it has been suggested that the early-life microbiota of the nasal mucosa is associated with the subsequent development of respiratory tract infections and allergic diseases [48,49]. The Growing Up in Singapore toward Healthy Outcomes (GUSTO) birth cohort study showed that bacterial diversity and the overall abundance of *Corynebacterium* spp. in the nasal microbiota were enriched in healthy infants compared to those with rhinitis and/or wheeze in the first 18 months of life [44]. GUSTO follow-up data showed that 20% of infants with rhinitis persisted with rhinitis at 5 years of age, while controls remained rhinitis-free indicating that rhinitis status at 18 months is a predictor of rhinitis status at 5 years [44]. In particular, *Corynebacterium* spp. in the nasal epithelium of healthy infants has been shown to inhibit *S. aureus* colonization, which in turn reduces *S. aureus* infections [50]. From an epigenetic perspective, Morin et al. found that upper airway microbiota diversity at 1 week of age was associated with AR risk at 6 years of age [18]. Multiple studies have also shown that increased abundance of *M. catarrhalis* and *H. influenzae* (*phylum Proteobacteria*) and *S. pneumoniae* (*phylum Firmicutes*) in the upper airway tract (UAT) microbiome of early infancy is associated with the subsequent risk of developing persistent childhood wheeze disorders [49,51,52,53]. McDade et al. further confirmed that early-life microbial exposure (e.g., contact with animal feces, experience of diarrhea) calibrates the body’s immune-inflammatory regulatory function, thereby reducing susceptibility to allergic inflammatory diseases in adulthood [54]. Additionally, rich microbial exposure in early life—such as exposure to farm environment microbes (e.g., *Acinetobacter lwoffii*) and consumption of raw milk—can promote the activation of regulatory T cells (Treg) through epigenetic modifications (e.g., demethylation of the FOXP3 gene). Alternatively, it can regulate the balance of innate lymphoid cell (ILC) subsets via short-chain fatty acids (SCFAs), metabolites of the gut microbiota. These processes further reduce the risk of allergic diseases [55], providing immunological evidence for the mechanism by which “early microbial exposure influences the development of allergic diseases.” These results support the predictive role of early upper respiratory tract microbial composition in subsequent allergic diseases. Therefore, it is crucial to clarify the full spectrum of nasal mucosal microbiome changes—especially the molecular mechanisms by which it regulates the expression of immune-related genes through epigenetic modifications. This is essential for identifying potential biomarkers for allergic rhinitis (AR) diagnosis and developing effective therapeutic strategies. However, research on the relationship between nasal microbiome dysbiosis and the development of allergic diseases in children and adolescents remains limited. Further studies are needed to confirm this association.

### 4.2. Characteristics of the Nasal Tract Microbiome in Adults with Allergic Rhinitis

Accumulating evidence suggests that the nasal mucosal microbiome may play an important role in the alteration of immune responses and the development of AR [45]. The upper respiratory tract consists of distinct anatomical structures, possesses diverse epithelial cell types, and is exposed to various environmental factors—leading to potential abnormalities in the nasal microbiota, particularly in the context of respiratory allergies [56]. A report concluded that *Staphylococcus*, *Propionibacterium*, *Corynebacterium*, and *Streptococcus* are the common bacterial genera in the nasal mucosa of patients with allergic rhinitis (AR) [57]. With the advancement of high-throughput sequencing techniques, some researchers have provided data suggesting nasal microbiota dysbiosis in adults with airway allergic inflammation [35]. Devyani et al. performed an interindividual microbiome analysis involving 65 participants and observed that the abundance of *S. aureus*, *Propionibacterium*, *Corynebacterium*, and *Peptoniphilus* in the nasal mucosa of AR patients is markedly elevated relative to healthy counterparts, whereas the abundance of *Prevotella* and *Streptococcus* is reduced [16]. Lee et al. noted that *Corynebacterium*, *Propionibacterium*, *Staphylococcus*, and *Streptococcus* represent predominant bacterial genera in the nasal mucosa of AR patients when contrasted with healthy controls [40]. Yau et al. established that *Staphylococcus* spp. are more prevalent in the nasal mucosa of patients with allergic rhinoconjunctivitis (ARC) [41]. Hyun et al. performed 454 pyrosequencing of the V1-V2 regions of the 16S rRNA gene on inferior turbinate mucosal biopsy samples from 32 subjects (20 in the AR group and 12 in the healthy control group). They identified a significant correlation between high total IgE levels (≥200 IU/mL) and inferior turbinate mucosal dysbiosis. The high IgE group showed a marked increase in the relative abundance of *Staphylococcus aureus* and a decrease in *Propionibacterium acnes*. Additionally, the microbial diversity (Shannon index) in this group was 23% lower than that in the control group (*p* < 0.01). This result provided key evidence for the notion that “*S. aureus* may serve as a potential microbial biomarker for the high IgE status in AR patients.” It should be noted that this study had certain methodological limitations: the sample size was small (total *n* = 32) and it lacked further stratified analysis of the impact of allergen exposure types (e.g., dust mites, pollen) on the microbiota [58]. Future studies could further verify the association specificity between *S. aureus* and AR-related immune markers (e.g., IL-4, IL-13), providing more precise evidence for the microbiota-AR causal mechanism. Due to the lack of large-scale, standardized, or uniformly annotated nasal microbiota detection, the results of different cross-sectional studies vary [59] (Table 2).

### 4.3. Regulatory Effects of Environmental and Clinical Factors on Nasal Microbiota Dysbiosis in Allergic Rhinitis

It is noteworthy that the composition of the nasal microbiota may also be significantly regulated by clinical interventions, the rhythm of allergen exposure, and external environmental factors. Commonly used therapeutic drugs for AR (e.g., intranasal glucocorticoids, oral antihistamines) can alter the microbial balance by inhibiting local inflammation or directly affecting microbial colonization. A retrospective study by Wu et al. involving 202 AR patients showed that among 100 patients receiving conventional treatment (mometasone furoate nasal spray + loratadine), the abundance of biofilm-forming *Staphylococcus aureus* (*S. aureus*) in the nasal cavity decreased significantly after 1 month of treatment (*p* < 0.001), and the biofilm level of *Staphylococcus epidermidis* (*S. epidermidis*) also decreased significantly (*p* = 0.001). This suggests that drug treatment may partially reverse the nasal microbiota dysbiosis in AR patients by directly inhibiting the proliferation of pathogenic bacteria—for example, reducing the proportion of pathogenic bacteria related to biofilm formation—which may further mask the original characteristics of microbiota dysbiosis in the untreated state [60]. Therefore, when analyzing the nasal microbiota data of AR patients, drug use history should be prioritized as a key confounding factor.

In addition, seasonal peaks of allergens such as dust mites and pollen are accompanied by fluctuations in the intensity of mucosal inflammation, which in turn affect the balance of microbial colonization. Although existing studies lack direct seasonal tracking data, there is evidence indicating the association between different allergen types and the specific microbiota: the aforementioned study by Chiu et al. found that the abundance of *Moraxella* in the nasal cavity of AR children sensitized to dust mites increased significantly to 1.95%, which was 3.9 times that of the healthy control group (0.50%) (FDR *p* = 0.030). *Moraxella* had a significant interaction with dust mite sensitization and the pathogenesis of AR, and it was a key microbiota involved in the occurrence of AR associated with dust mite exposure [42]. This finding directly supports the hypothesis that “specific allergen exposure is associated with abnormal microbiota abundance.” Manirajan et al. further confirmed that bacteria colonizing the surface of highly allergenic pollens (e.g., birch and hazelnut)—such as *Methylobacterium* and *Spirosoma*—can produce higher levels of endotoxins (LPS, LTA). These bacteria act together with pollens to affect the microorganisms colonizing the nasal cavity and induce respiratory epithelial cells to release pro-inflammatory factors such as IL-8 and TNF-α, which significantly enhance the seasonal allergic inflammatory response and also suggest that seasonal allergen exposure may regulate the composition of the nasal microbiota [61].

As an important external environmental influencing factor, air pollution can change the nasal microenvironment of patients with allergic rhinitis. A study by Yang et al. on 1121 preschool children in Taiyuan, China further confirmed that under high exposure to PM_2.5_, SO_2_, and O_3_, the abundance of risky bacteria (e.g., *Amaricoccus*, *Blautia*) in the nasal cavity increased, while the abundance of protective bacteria (e.g., *Dolosigranulum*) decreased, and the characteristics of microbiota dysbiosis were more significant [62]. In addition, nicotine and polycyclic aromatic hydrocarbons in tobacco smoke can directly inhibit the colonization of beneficial bacteria and amplify microbiota dysbiosis. A pilot study by Brindisi et al. on AR children found that passive smoking exposure was closely related to the disruption of nasal microbiota homeostasis—compared with non-exposed children, AR children exposed to passive smoking had significantly lower abundance of beneficial bacteria (e.g., *Aggregatibacter*, *Propionibacterium*) and a higher colonization risk of pathogenic bacteria such as *Staphylococcus aureus*, and this microbiota dysbiosis phenotype was positively correlated with the severity of AR symptoms [61]. Further research by Yang et al. confirmed that in children exposed to environmental tobacco smoke (ETS), the abundance of Haemophilus, which has immunomodulatory effects in the nasal cavity, decreased, which indirectly weakened the inhibitory effect on microbiota dysbiosis and aggravated AR-related inflammation [62].

However, most existing studies adopt a cross-sectional design, and there is no standardized control over the seasonal cycle and exposure intensity of allergens. There is also a lack of longitudinal tracking of the “seasonal exposure—microbiota change—inflammation fluctuation” triad, which may lead to biases in the interpretation of the association between microbiota characteristics and allergens. Moreover, the control of drug use duration and types is insufficient (e.g., intranasal glucocorticoids may alter the abundance of pathogenic bacteria), which may interfere with the true presentation of microbiota characteristics. These are the key directions that need to be addressed and improved in future studies.

**Table 2:** Changes in Nasal Microbiota in Adult Patients with Allergic Rhinitis.

Population	Sample Site	Actinobacteria	Bacteroidetes	Firmicutes	Proteobacteria	Study
Adults with AR	Middle meatus	*↑* *Propionibacterium*		*↑* *parvimonas* *↑* *Staphylococcus* *↑* *Lactococcus* *↑* *Enterococcus*	*↓* *Ralstonia* *↑* *unclassified* *Enterobacteriaceae*	[16,63]
Adults with AR	Inferior turbinate			*↑* *Pseudomonas* *↓* *Serratia* *↓* *Ralstonia*		[64]
Adults with AR + asthma	Nasal lavage solution		*↓* *Prevotella* *↓* *Rothia*	*↑* *Faecalibacterium* *↑* *lactobacillus* *↑* *clostridium_IV* *↑* *blautia* *↑* *butyricicoccus*	*↑* *Escherichia* *↑* *pelomonas*	[65]
Adults with AR	Nasal extracellular vesicles			*↑* *Acetobacter* *↓* *Streptococcus*	*↑* *Escherichia* *↑* *Halomonas* *↓* *Zoogloea* *↓* *Burkholderia* *↓* *Pseudomonas*	[43]

Note: The upward arrow (↑) represents an increased relative abundance of the corresponding microbial taxa, and the downward arrow (↓) represents a decreased relative abundance of the corresponding microbial taxa.

## 5. Potential Mechanisms Linking URT Microbiome to Allergic Rhinitis

### 5.1. The Role of Bacteria

The incidence of allergic diseases is closely associated with the interactions between host systems and resident microbiota. When the integrity of the epithelial barrier is impaired, the composition of microbial communities may undergo alterations, thereby triggering inflammatory, allergic, and autoimmune responses [66,67]. Nasal epithelial cells serve as the primary target for environmental allergens, and recent studies have underscored their pivotal role as a barrier restricting host contact with allergens in allergic rhinitis (AR). The nasal microbiome resides in nasal mucus, interfaces with inhaled pathogens or allergens, and may undergo functional crosstalk with the nasal epithelium, notably in the regulation of immune mechanisms within the upper respiratory tract [63].

Miao et al. (Ping Miao) identified through 16S rRNA sequencing that, compared with healthy individuals (*n* = 105), the abundance of *Streptococcus salivarius* in the nasal microbiome of patients with allergic rhinitis (AR, *n* = 55) was increased. Using in vitro and in vivo AR models, they confirmed that this commensal bacterium promoted the release of inflammatory cytokines and induced morphological changes in nasal epithelial cells, thereby driving the occurrence and progression of AR [68]. In a study exploring the mechanism by which *Streptococcus salivarius* stimulates cytokine responses during AR (allergic rhinitis), on the one hand, the researchers found through experiments on a mouse model of airway inflammation induced by *Alternaria alternata* (*A. alternata*, a clinical AR-related environmental allergen) that treatment with either *A. alternata* alone or *Streptococcus salivarius* (isolated from the nasal cavity of AR patients) alone could upregulate the expression of multiple cytokine genes; moreover, the mRNA levels of inflammatory cytokines (IL-1β [69], IL-6 [70], TNF-α [71]), epithelial cytokines (IL-25 [72]) and type 2 immune response-related cytokines (IL-5) in mice co-treated with both microorganisms were significantly higher than those in mice treated with a single microorganism [73]. On the other hand, ex vivo experiments analyzed the effects of 5 randomly selected nasal isolates of *Streptococcus salivarius* (from AR patients) and 5 randomly selected nasal isolates of *Staphylococcus epidermidis* (as controls) on allergen-exposed airway epithelial cell (A549) monolayers. The results showed that all *Streptococcus salivarius* isolates (whereas *Staphylococcus* epidermidis isolates had no such effect) could stimulate the expression of pro-inflammatory cytokines (IL-6, IL-8, TNF-α), epithelial cytokines (IL-33 and TSLP, which can trigger type 2 immune cell-mediated allergic cascades), and the chemokine eotaxin-1 (CCL11)—an eosinophil-specific chemoattractant (eosinophils possess allergy-related characteristics). Therefore, the above findings indicate that nasal isolates of *Streptococcus salivarius* have a specific ability to induce cytokine expression in allergen-exposed nasal epithelial cells and may be involved in the pathophysiological processes of AR [68].

**Figure 1:**
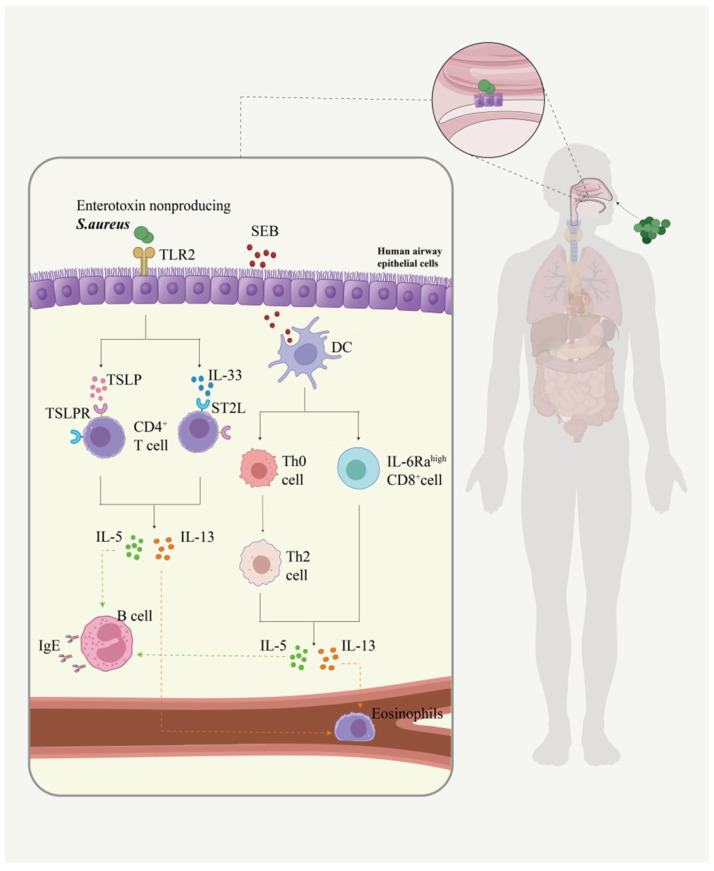
The mechanism of action by which *Staphylococcus aureus* induces allergic rhinitis.

Notably, as another bacterium with abnormal abundance in the nasal microbiome of AR patients, the regulatory mechanism of *Staphylococcus aureus* on AR differs from that of *Streptococcus salivarius* (which acts synergistically with allergens such as *A. alternata*). Instead, *Staphylococcus aureus* disrupts nasal immune homeostasis through innate immune activation and superantigen effects. Data from a 2016 study showed that after binding to Toll-like receptor (TLR2), *Staphylococcus aureus* (*S. aureus*) induces the production of type 2 cytokines (e.g., IL-5 and IL-13) via IL-33 and thymic stromal lymphopoietin (TSLP) released from human airway epithelial cells [44]. The effects of staphylococcal enterotoxin A (SEA) are biased toward ancillary functions, and their correlation with AR remains unclear [74]. Furthermore, staphylococcal enterotoxin B (SEB) exerts substantial effects on the inflammatory response. SEB first activates innate immune cells: it induces dendritic cells (DCs) maturation and enables activated DCs to drive naive T cells polarization toward the type 2 immune phenotype, which promotes the acute-phase atopic profile of AR patients [75]. Superantigens such as enterotoxins directly stimulate mast cell (MC) degranulation, triggering the release of histamine, IL-6, IL-8, prostaglandin D2, TNF-α, IL-23, and IL-31. Together with TSLP released from damaged nasal epithelial cells, these mediators exacerbate the type 2 inflammatory response [75,76]. Staphylococcal enterotoxin B (SEB) promotes the secretion of IL-5 and IL-13 through its effects on type 2 immune cells [77]. IL-5 is involved in the recruitment, maturation, and activation of eosinophils [78]. IL-13 upregulates class II expression in B cells and promotes IgE class switching; IgE then binds to mast cell receptors [79] (Figure 1). The above evidence supports the idea that *S. aureus* induces IgE production and promotes allergic inflammation. Thus, controlling the levels of both S. aureus and IgE may be an effective strategy for preventing IgE-associated diseases, including AR (Figure 1).

In addition, another study enrolled 32 AR patients and 20 healthy controls, who underwent 16S rDNA sequencing and untargeted metabolomics analysis to compare microbial diversity, composition, and functional pathways between the groups. The results showed that compared with the control group, the relative abundance of *Actinobacteria* in the AR group was significantly decreased, while that of *Bacteroidetes* was significantly increased (*p* < 0.05), with significant enrichment of *Vibrio* and *Aeromonas*. Metabolomics analysis identified 528 differential metabolites, and correlation analysis revealed significant associations between specific microbial taxa (e.g., Aeromonas, Vibrio) and metabolites (e.g., LPC, arachidonic acid)—indicating a potential link between microbiota-derived metabolic changes and inflammatory responses in AR [80]. Notably, a recent study has confirmed that microbiota-metabolite interactions (e.g., LPC-related) influence the occurrence of dysbiosis and inflammation via the TLR4/NF-κB signaling pathway [80]. LPC can serve as a biomarker for the severity of inflammation. This lipid molecule activates and regulates the migration of immune cells via G protein-coupled receptors, and induces the release of inflammatory factors, as well as oxidative stress and cell death. These processes collectively exacerbate inflammation, drive disease progression, and further aggravate nasal mucosal inflammation [81].

In contrast to the pro-inflammatory effects of pathogenic bacteria such as *Staphylococcus aureus* and *Streptococcus salivarius*, core nasal protective bacteria including *Corynebacterium accolens* and *Dolosigranulum pigrum* are involved in maintaining nasal microecological homeostasis. As a nasal protective bacterium, the ameliorative effect of *C. accolens* on AR-related microbiota dysbiosis has been verified through multiple research dimensions. De Steenhuijsen Piters et al. identified through molecular epidemiology and mechanism exploration that C. accolens can utilize triglycerides (TAGs) on the surface of the host’s nasal mucosa and skin. It releases free fatty acids (FFAs) such as oleic acid and linoleic acid via hydrolysis, and these FFAs possess direct anti-pathogenic activity, significantly inhibiting the growth of potential pathogens like *Streptococcus pneumoniae*. Meanwhile, this bacterium often colonizes the nasal cavity synergistically with *Dolosigranulum* spp.; their coexistence further reduces pathogenic bacterial abundance and the risk of acute respiratory infections. Additionally, *C. accolens* can induce host sebaceous gland cells to secrete human β-defensin 2 (HBD-2), indirectly preventing pathogen invasion by enhancing mucosal innate immune defense [82]. Subsequent research by Menberu et al. supplemented key evidence from the perspectives of strain function and in vitro/in vivo validation. *C. accolens* strains (C779, C781, C787) isolated from the nasal cavity of healthy individuals exhibited an adhesion rate of 50–70% to human nasal epithelial cells (HNECs), with C781 showing the strongest adhesion ability. These strains significantly inhibited the adhesion (inhibition rate: 24–50%) and biofilm formation of *S. aureus* (including MRSA) on HNECs by competing for adhesion sites and nutrients, without exerting cytotoxicity on HNECs (no significant difference in lactate dehydrogenase (LDH) release compared with the control group). More importantly, although *C. accolens* itself does not induce IL-6 secretion, it significantly reduces *S. aureus*-induced pro-inflammatory factor IL-6 release (*p* < 0.0001, with strain C787 demonstrating the optimal anti-inflammatory effect) [83], This indicates that *C. accolens* has a negative correlation with *S. aureus* and is indirectly involved in regulating nasal microecological balance, further highlighting its core value in correcting AR-related microbiota dysbiosis. As a key protective bacterium that colonizes synergistically with *C. accolens*, the anti-pathogenic effect of *Dolosigranulum pigrum* has also been confirmed by the latest research. Cole et al. conducted a 5-month longitudinal nasal sampling of 31 healthy subjects and in vitro functional experiments. They found that *D. pigrum* was only detected in the nasal cavity of individuals with intermittent or no *Staphylococcus aureus* (*SA*) colonization, but not in those with persistent *SA* colonization. In vitro simulation of the nasal mucosal environment showed that *D. pigrum* reduced the recovery rate of *SA* to below 10%, significantly inhibiting the adhesion and proliferation of *SA* on nasal mucosal epithelial cells. Moreover, the inhibitory effect on *SA* was more pronounced when *D. pigrum* acted synergistically with *C. accolens* compared with that of a single strain, further supporting their synergistic protective role in the nasal microecology [84]. Both *C. accolens* and *D. pigrum* maintain nasal microbiota balance through direct anti-pathogenic effects and synergistic microecological regulation. The aforementioned studies form a complete evidence chain covering metabolic antibacterial activity, competitive inhibition, mucosal barrier protection, and synergistic symbiosis. Although they do not directly confirm the association between these two bacteria and AR, when combined with the core characteristics of AR pathogenesis—impaired nasal mucosal barrier and enrichment of pathogenic bacteria such as *S. aureus*—it is suggested that these two protective bacteria may be indirectly involved in AR prevention and control by improving nasal microecological imbalance. This provides indirect scientific evidence for their use as candidate strains for AR microbial-targeted therapy.

Over 90% of current studies on the association between microbiota and AR adopt cross-sectional or case–control designs. Such designs can only reveal the correlation between the two, but cannot determine the temporal sequence of “microbiota dysbiosis preceding AR onset” nor rule out interference from reverse causation (e.g., AR inflammation itself alters the nasal microenvironment, thereby inducing microbiota dysbiosis) or confounding factors (e.g., drugs, environmental allergens). Only one prospective cohort study (GUSTO cohort) observed that “nasal microbiota characteristics of infants at 18 months of age were associated with AR development at 5 years of age” [44], but the causal relationship has not been verified through intervention. Therefore, more studies are needed in the future to clarify the direction of the association between microbiota and AR.

*Staphylococcus aureus* binds to Toll-like receptor 2 (TLR2) on airway epithelial cells, prompting the release of interleukin-33 (IL-33) and thymic stromal lymphopoietin (TSLP), which in turn induce the production of T helper 2 (Th2)-type cytokines such as IL-5 and IL-13. Toxins secreted by *Staphylococcus aureus*, including staphylococcal enterotoxin B (SEB), can activate dendritic cells and drive the polarization of naive T cells toward the Th2 phenotype. Additionally, these toxins can directly stimulate mast cell degranulation to release inflammatory mediators, which synergize with TSLP released by nasal mucosal epithelial cells to exacerbate Th2 inflammation. SEB can also directly induce Th2 cells to secrete IL-5 and IL-13: IL-5 regulates eosinophils, while IL-13 promotes immunoglobulin E (IgE) production by B cells. The binding of IgE to mast cells lays the foundation for the inflammatory response in allergic rhinitis (AR). The illustration was created using Adobe Illustrator 2023. Figure 1 was created using Adobe Illustrator 2023.

### 5.2. The Role of Viruses

**Figure 2:**
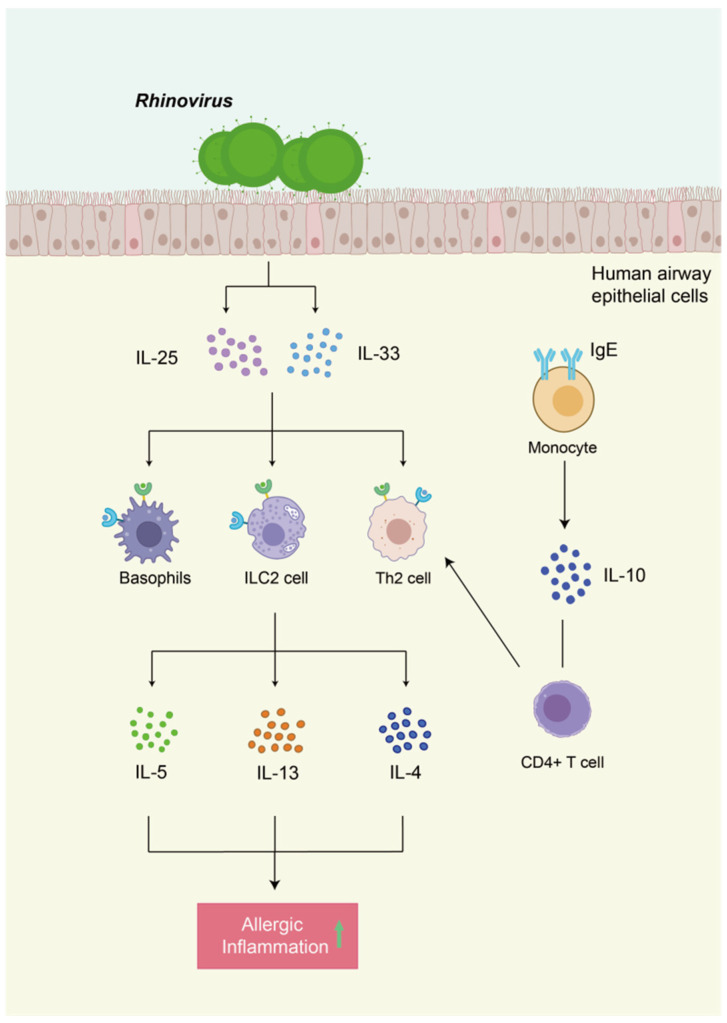
The mechanism by which rhinoviruses enhance allergic inflammation.

Studies on viruses in the nasal microbiome are relatively limited. However, viral infections may also play a role in allergic mechanisms. Vanders et al. found that Influenza A virus (IAV) infection in pregnant mice with allergic airway disease was associated with increased mucus hypersecretion, enhanced airway hyperresponsiveness (AHR), and aggravated allergic airway disease [85]. Studies by Gao et al. have demonstrated that the detection rate of human rhinovirus (HRV) is higher in children with allergic rhinitis (AR), and that HRV infections (species A, B, or C) are positively correlated with symptom severity in AR children [86]. In a controlled study, Ruiz et al. found that infants co-infected with respiratory syncytial virus (RSV) and rhinovirus (HRV) before the age of two were more likely to develop asthma at seven to eight years of age, while a single HRV infection was associated with a higher risk of developing allergic rhinitis [87]. Rhinovirus—one of the most common viruses in the human respiratory tract—is closely related to the occurrence and development of allergic asthma and plays a key role in promoting the type 2 immune response. IL-25 and IL-33 are produced by human respiratory epithelial cells stimulated by rhinoviruses, which then bind to receptors on Th2 cells, ILC2s, and basophils to drive the production of IL-4, IL-5, and IL-13 [86,88]. Furthermore, in the study conducted by Rigina et al., it was found that IgE-mediated monocyte stimulation enhances rhinovirus (RV)-driven naive CD4^+^ T cell differentiation into T helper 2 (Th2) cells, and this process is regulated by the inhibition of both IgE-induced interleukin-10 (IL-10) production and virus-induced type I interferons. Specifically, the interplay between IgE-driven allergic stimulation and rhinovirus (RV) exposure promotes enhanced Th2 cell differentiation via IgE-elicited effects on monocytes. Without IgE-mediated signaling, Th2 differentiation is modulated by virus-derived type I interferons, ultimately yielding a predominantly T helper 1 (Th1)-skewed antiviral response. However, under conditions of IgE-mediated stimulation, IgE-induced IL-10 production suppresses the response of monocyte-derived interferons to RV infection. When combined with other uncharacterized IgE-mediated factors—such as altered expression of co-stimulatory molecules, cytokines, and chemokines—this suppression enables the increased differentiation of Th2 cells. In vivo, allergic inflammation may be exacerbated during infection due to the loss of interferon-induced negative regulation on Th2 cell development. This mechanism reveals a critical immunoregulatory pathway underlying the exacerbation of allergic diseases (e.g., allergic rhinitis) by rhinoviruses [89]. The above evidence suggests that it is important to further understand the role of viruses in allergic diseases and the changes in immune status after infection (Figure 2).

After being stimulated by rhinoviruses, human respiratory epithelial cells produce interleukin-25 (IL-25) and interleukin-33 (IL-33). These two cytokines then bind to receptors on the surface of T helper 2 (Th2) cells, type 2 innate lymphoid cells (ILC2s), and basophils, thereby inducing these cells to produce IL-4, IL-5, and IL-13. And the cross-linking of IgE and exposure to rhinoviruses mutually reinforce each other, synergistically promoting the differentiation of naive CD4+ T cells into Th2 cells. Figure 2 was created using Adobe Illustrator 2023.

### 5.3. The Roles of Other Microorganisms

Furthermore, in addition to bacteria and viruses, fungi are also involved. Fungi are widespread eukaryotic microbes frequently linked to numerous disorders. Exposure to fungal bioaerosols often correlates with conditions including asthma, AR, keratitis, and pneumonia. Fungal conidia are generally less than 10 μm in size, enabling them to reach the nasal sinuses and paranasal sinuses as well as the lower respiratory tract, where fungal inflammation may occur [90]. Fungal spores are ubiquitous, and the number of fungal species present in the environment is estimated to be at least one million. Several genera of airborne fungal spores, such as *Alternaria*, *Aspergillus*, and *Cladosporium*, are found worldwide; the airborne spores of these fungi are generally recognized as important causative agents of allergic rhinitis (AR) and allergic asthma [91]. Currently, research on the mechanisms by which fungi induce allergic rhinitis (AR) remains limited. However, existing studies have demonstrated that fungi (e.g., *Aspergillus*, *Alternaria*) can disrupt epithelial junctions through the release of proteases. For instance, the Alp1 protease degrades tight junction proteins (such as occludin) in the airway epithelium, which increases barrier permeability and facilitates the entry of allergens into the submucosa, thereby enhancing allergen penetration [92]. Further research is required to confirm the specific mechanisms underlying the role of fungi and other microorganisms in allergic rhinitis (AR).

### 5.4. The Role and Potential of the Gut-Lung Axis and Gut-Nasal Axis in Nasal Microbiota Dysbiosis and Allergic Rhinitis (AR)

Over the past 20 years, research on the relationship between the gut microbiota and host metabolism and immune responses has remained a hot topic [93]. In recent years, the novel concept of the “gut-lung axis” has been proposed: in addition to the local environment, the gut microbiota can also exert a key regulatory role in respiratory immune responses, a process possibly mediated by the systemic diffusion of metabolites through the bloodstream [94,95]. The gut and lungs are connected through direct and indirect pathways. Direct pathways include swallowing infected sputum or inhaling gastroesophageal contents. Indirect pathways are mediated by immune signaling and the systemic spread of microbial metabolites, cytokines, and bacterial fragments, which can enter the bloodstream and regulate distant immune regions including the lungs [96]. In AR-related studies, Watts et al. found that adult AR patients had reduced gut microbiota diversity, with a significantly lower abundance of Firmicutes compared to healthy individuals [97]. Zhou MS et al. reached the same conclusion and further observed that AR patients had significantly lower concentrations of short-chain fatty acids (SCFAs) than healthy controls [98]. However, a study on an AR mouse model showed opposite changes in the abundance of specific bacterial taxa. Nevertheless, the research team found that α-linolenic acid (ALA) levels in the feces and serum of AR mice were consistently reduced [93]. Both ALA and SCFAs are metabolized from food by the gut microbiota and are beneficial for maintaining immune homeostasis. ALA can be converted to docosahexaenoic acid (DHA), which in turn reduces prostaglandin E synthesis and inhibits the production of allergy-related cytokines and immunoglobulin E (IgE) [99]. Crosstalk may also exist between the gut microbiota and upper airway tract (UAT) microbiota. For example, changes in the neonatal diet can affect the composition of the lung microbiota, and fecal microbiota transplantation in rats can induce alterations in the lung microbiota [100,101]. However, research on the impact of the respiratory microbiota on the gut microbiota still requires further exploration. In a study on *Helicobacter pylori*, positive Mendelian randomization analysis revealed that *H. pylori* infection may influence the occurrence and development of allergic diseases through a complex network involving the “gut-lung axis” and “gut-nasal axis.” The results indicated that outer membrane protein (OMP) antibodies may be protective factors against AR. Additionally, accumulating evidence suggests the existence of the “gut-nasal axis,” which regulates microbiota-immune interactions through information exchange across mucosal surfaces. As one of the most abundant metabolites of the gut microbiota, SCFAs exert anti-inflammatory effects by inhibiting T helper 17 (Th17) cells, regulating the secretion of cytokines such as interleukin-4 (IL-4), interleukin-5 (IL-5), and interleukin-10 (IL-10), reducing the activation of dendritic cells (DCs), and increasing the number of regulatory T cells (Treg). The upregulation of OMP antibodies may activate these anti-inflammatory signaling pathways through SCFAs, thereby exerting immunomodulatory effects and potentially reducing susceptibility to AR. This process also enables the gut microbiota to remotely regulate the nasal microbiota [59].

The limitations of current research are mainly reflected in three aspects. First, direct evidence is insufficient: most studies only observe correlations between the gut and nasal microbiota, lacking causal verification of the gut-nasal axis. Second, the mechanisms are unclear: the specific signaling pathways and key molecular targets through which the gut microbiota regulates the nasal microbiota have not been elucidated. Third, intervention studies are scarce: AR prevention and treatment strategies based on the gut-nasal axis (e.g., gut probiotic intervention) are still in the preliminary stage, and their efficacy in improving nasal microbiota dysbiosis and dose–response relationships remains unclear. Future studies should adopt multi-omics combined analysis and clinical intervention experiments to further explore the regulatory potential of the gut-nasal axis, providing new theoretical basis and technical approaches for microecology-targeted therapy of AR.

## 6. Modulation of the Nasal Microbiota May Serve as a Novel Therapeutic Approach for Allergic Rhinitis

Based on the regulatory mechanisms of nasal microbiome dysbiosis in allergic rhinitis (AR) as described earlier, the targeted correction of such dysbiosis represents a novel potential therapeutic strategy for allergic rhinitis (AR). As core modulators, probiotics, by reshaping the microbiota, have shown promising progress in their application as adjuvant therapy for allergic rhinitis. Most commonly used probiotic products include *Lactobacillus*, *Bifidobacterium*, *Lactococcus*, *Streptococcus* and *Enterococcus* [102]. International Consensus Statement on Allergy and Rhinology: Allergic Rhinitis recommends considering probiotics as an adjuvant therapy for patients with AR due to their low risk and proven efficacy in improving symptoms [103].

The administration routes of probiotics include oral administration and local (nasal) administration. Currently, research on oral probiotics is relatively widespread. However, the mechanisms involved in oral probiotics may include systemic effects on the immune system rather than locally initiated actions—such as the stabilization of nasal epithelium and the reduction of pro-inflammatory cytokine release. Therefore, compared with the main drugs for the treatment of allergic rhinitis (AR) (i.e., corticosteroids and antihistamines), oral probiotics appear to exert a relatively weak effect, with potentially limited clinical relevance. An intranasal instillation experiment of probiotic extracts in mice demonstrated that *Clostridium butyricum* extracts can inhibit allergic rhinitis by upregulating interleukin-10 (IL-10), providing a theoretical basis for the local therapeutic application of probiotics [104]. Moreover, in a mouse model of AR, *Lactobacillus rhamnosus (L. rhamnosus)* was suggested to attenuate allergen-induced production of type-2 cytokines (IL-5 and IL-13) and reduce allergen responsiveness [105,106]. The potential of intranasal administration of the probiotic *L. rhamnosus* GG (LGG strain) has been fully validated in animal experiments. Spacova et al. administered the LGG strain intranasally at a dose of 5 × 10^8^ CFU per administration, which prevented birch pollen-induced allergic asthma [106]. Furthermore, a study by Pellaton et al. confirmed that intranasal administration of *Lactobacillus paracasei* NCC2461 at 1 × 10^9^ CFU per administration reduced the number of eosinophils in bronchoalveolar lavage fluid (BALF) by 37.8-fold (*p* < 0.0005), significantly decreased IL-5 and eosinophil chemotactic factor levels in lung tissue, and the efficacy of intranasal administration was significantly superior to that of intragastric administration [105]. A randomized controlled trial (RCT) evaluated a probiotic nasal irrigation solution containing *Lactobacillus plantarum* and *Bifidobacterium animalis*. The results showed a significant reduction in the abundance of pathogenic bacteria (e.g., *Haemophilus* and *Staphylococcus*) in the nasal cavity, accompanied by an increase in probiotic colonization. However, it should be noted that intranasal probiotic administration did not improve AR symptoms (e.g., Total Nasal Symptom Score [TNSS], Mini-Rhinoconjunctivitis Quality of Life Questionnaire [Mini-RQLQ]), which may be related to strain selection, intervention dose, or individual patient differences [107]. Furthermore, a meta-analysis incorporating 28 randomized controlled trials (RCTs) demonstrated that probiotics significantly reduced the nasal and ocular symptom scores (standardized mean difference [SMD] = −1.23, *p* < 0.001), with particularly notable effects observed in seasonal rhinitis and for the *Lactobacillus paracasei* LP-33 strain [108]. However, the method of intranasal administration is still controversial, and additional research is required in the future to provide evidence for its validity.

Notably, this therapeutic strategy may also be applicable to local allergic rhinitis (LAR)—a subtype of rhinitis that has received increasing attention in recent years, and whose pathogenesis may also be closely related to nasal microbiome dysbiosis. The core characteristics of LAR include typical AR symptoms (e.g., rhinorrhea, sneezing, nasal itching) triggered by exposure to airborne allergens such as dust mites, grass pollen, and *Alternaria alternata*, while conventional systemic atopy tests (skin prick tests [SPT], serum specific IgE test) are negative [109]. Mechanistically, LAR is centered on local type 2 inflammation of the nasal mucosa, involving mast cell activation, eosinophil infiltration, and local allergen-specific IgE production. This characteristic of local immune imbalance suggests a potential association with nasal microbiome dysbiosis [110]. Similar to the microbiome dysbiosis pattern in classic AR—“reduced abundance of beneficial bacteria (e.g., *Corynebacterium*, *Dolosigranulum*) and overproliferation of pathogenic bacteria (e.g., *Staphylococcus aureus*)”—the specific pattern of nasal microbiome abnormalities in LAR requires confirmation through more targeted studies.

Furthermore, the clinical translation of probiotics in diseases such as allergic rhinitis (AR) faces multiple core limitations. First, probiotic colonization is highly dependent on the adaptability of the host’s nasal microenvironment. Bourdillon et al. noted that native nasal microbiota (e.g., *Corynebacterium*, *Dolosigranulum*) can interact with exogenous probiotics, impairing their adhesion and resulting in insufficient intervention response rates in some AR patients. Second, administration methods significantly affect efficacy: local nasal delivery (e.g., nasal spray, irrigation) yields more stable effects than oral administration by acting directly on the nasal mucosa [111]. A meta-analysis by Luo et al. supplemented that oral probiotics must pass through the gastrointestinal tract, where gastric acid and digestive enzymes inactivate some strains—only a few highly tolerant strains can exert biological effects. The regulatory effect of oral probiotics on the nasal microecology is highly heterogeneous (I^2^ = 89–97%), and significant differences in efficacy are observed due to variations in AR patients’ age, disease subtype (seasonal AR [SAR]/perennial AR [PAR]), and environmental exposure [108]. Children and adults also differ in their tolerance and responsiveness to probiotics [112,113], but dedicated studies on children are relatively scarce, with insufficient supporting evidence. Although no serious adverse reactions have been reported in probiotic therapy for AR, mild gastrointestinal symptoms such as diarrhea and abdominal pain have been documented in some randomized controlled trials (RCTs) [114,115]. Bourdillon et al. also warned of potential probiotic infection risks (e.g., bacteremia) in immunocompromised populations. Additionally, some strains carry antibiotic resistance genes, and data on the risk of gene transfer during long-term intervention are lacking. Most existing studies have an intervention duration of 2–12 weeks, with no available data on colonization stability beyond 1 year [111]. Future research should focus on clarifying the compatibility between strains, doses, and populations, optimizing local nasal drug delivery methods, and conducting large-sample, long-term follow-up RCTs with unified evaluation criteria. It is also necessary to strengthen safety monitoring and antibiotic resistance risk assessment in special populations, while deepening studies on the interaction between probiotics and nasal microbiota as well as underlying immunomodulatory mechanisms—so as to promote the standardized clinical application of probiotics.

## 7. Limitations

It should be noted that the current review has certain limitations in perspective First, most included studies focus on specific populations (e.g., children in certain regions, adult patients with common allergens) and lack unified data from global multi-ethnic and multi-environmental cohorts, making it difficult to generalize the conclusions to broader populations. Second, the research methods are heterogeneous—differences in sampling sites (anterior nares, middle meatus, inferior turbinate), sequencing technologies, and analytical standards lead to inconsistencies in microbiota characterization results, hindering direct cross-study comparisons. Third, the mechanistic discussion is predominantly centered on bacterial taxa, with insufficient attention paid to the roles of viruses, fungi, and their interactions with bacteria in AR pathogenesis. Additionally, the understanding of the gut-nasal axis remains largely based on correlational evidence, lacking rigorous causal verification and clarification of key regulatory molecules. Finally, the exploration of microbiota-targeted therapies is limited by inconsistent intervention strategies (strain selection, dosage, course of treatment) and insufficient long-term follow-up data, making it challenging to establish standardized clinical application guidelines. These limitations highlight the need for more standardized, multi-center, and multi-omics integrated studies to fill the existing research gaps.

## 8. Conclusions

In conclusion, nasal microbiota dysbiosis is a key link in the pathogenesis of allergic rhinitis (AR), characterized by alterations in microbial diversity, disturbances in microbial community structure, and abnormalities in functional metabolism. This article reviews the latest research progress on the association between the nasal microbiota and AR, and further reveals the specific mechanisms by which the nasal microbiota regulates AR. Bacteria represented by *Staphylococcus aureus* and *Streptococcus salivarius* play critical roles; meanwhile, viruses such as rhinoviruses and fungi such as *Alternaria alternata* also participate in the AR process by regulating immune responses and disrupting the mucosal barrier. These findings supplement the understanding of the multi-microbial pathogenic mechanisms of AR, aiming to provide new insights for the research and treatment of AR. Finally, this article proposes novel AR therapeutic strategies targeting the modulation of nasal microbiota. The nasal microbiota serves as an important “regulator” in the occurrence and development of AR; future studies should consider integrating multi-omics technologies to clarify microbe-host interactions, thereby opening up new avenues for the precise prevention and treatment of AR.

## Figures and Tables

**Table 1:** The Composition of Nasal Microbiota in Healthy Individuals Across Different Age Groups.

Healthy Population	Sampling Site and Collection Method	Identification Techniques	Detected Bacteria	Study
**Infants**	Anterior nares, Nasal swab	16S rDNA gene sequencing	*Corynebacterium*, *Propionibacterium*, *Bifidobacterium*, *Streptococcus*, *Staphylococcus*, *Dolosigranulum*, *Moraxella*	[33]
	Nasopharynx, Nasopharyngeal swab	16S rDNA gene sequencing	*Corynebacterium*, *Propionibacterium*, *Bifidobacterium*, *Bacteroides*, *Staphylococcus*, *Faecalibacterium*, *Streptococcus*, *Moraxella*	[33]
	Oropharynx, Oropharyngeal swab	16S rDNA gene sequencing	*Prevotella*, *Streptococcus*, *Vaillonella*, *Haemophilus*, *Moraxella*, *Neisseria*	[33]
**Adults**	Anterior nares, Nasal swab	16S rDNA pyrosequencing; bacterial culture	*Corynebacterium*, *Propionibacterium*, *Prevotella*, *Dolosigranulum*, *Staphylococcus*, *Streptococcus*, *Moraxella*, *Escherichia shigella*	[24]
	Middle meatus, Middle meatus swab	16S rDNA pyrosequencing; bacterial culture; 16S rRNA and ITS next-generation sequencing	*Corynebacterium*, *Propionibacterium*, *Prevotella*, *Dolosigranulum*, *Staphylococcus*, *Streptococcus*, *Moraxella*, *Escherichia shigella*	[24,37]
	Sinus swab	16S rDNA gene sequencing	*Corynebacterium*, *Propionibacterium*, *Prevotella*, *Staphylococcus*, *Anaerococcus*, *Peptoniphilus*, *Ralstonia*	[34,38]
	Nasopharynx, Nasopharyngeal swab	16S rRNA gene sequencing	*Corynebacterium*, *Propionibacterium*, *Bifidobacterium*, *Prevotella*, *Sphingobacterium*, *Staphylococcus*, *Faecalibacterium*, *Streptococcus*, *Pseudomonas*, *Haemophilus*	[35]
	Oropharynx, Oropharyngeal swab	16S rRNA pyrosequencing; bacterial culture	*Corynebacterium*, *Rothia*, *Prevotella*, *Porphyromonas*, *Streptococcus*, *Vaillonella*, *Haemophilus*, *Moraxella*	[35]

## Data Availability

No new data were created or analyzed in this study. Data sharing is not applicable to this article.

## Abbreviations

AHR	Airway Hyperresponsiveness
ALA	α-Linolenic Acid
AR	Allergic Rhinitis
ARC	Allergic Rhinoconjunctivitis
ASV	Amplicon Sequence Variant
CCL11	Chemokine Eotaxin-1
CFU	Colony-Forming Unit
CRS	Chronic Rhinosinusitis
DCs	Dendritic Cells
DHA	Docosahexaenoic Acid
FDR	False Discovery Rate
FEAST	Fast Expectation-Maximization for Microbial Source Tracking
FFAs	Free Fatty Acids
GUSTO	Growing Up in Singapore toward Healthy Outcomes
HDM	House Dust Mites
HNEC	Human Nasal Epithelial Cells
HRV	Human Rhinovirus
HBD-2	Human β-Defensin 2
IAV	Influenza A Virus
ILC2	Type 2 Innate Lymphoid Cells
LDA	Linear Discriminant Analysis
LDH	Lactate Dehydrogenase
LPS	Lipopolysaccharide
LPC	Lysophosphatidylcholine
LTA	Lipoteichoic Acid
MC	Mast Cell
Mini-RQLQ	Mini Rhinoconjunctivitis Quality of Life Questionnaire
MRSA	Methicillin-Resistant *Staphylococcus aureus*
OTU	Operational Taxonomic Unit
PAR	Perennial Allergic Rhinitis
RCT	Randomized Controlled Trial
RV	Rhinovirus
RSV	Respiratory Syncytial Virus
SCFAs	Short-Chain Fatty Acids
SEA	Staphylococcal Enterotoxin A
SEB	Staphylococcal Enterotoxin B
SA	*Staphylococcus aureus*
SAR	Seasonal Allergic Rhinitis
SMD	Standardized Mean Difference
SPT	Skin Prick Test
TAGs	Triacylglycerols
Th1	T Helper 1 Cells
Th2	T Helper 2 Cells
TLR2	Toll-Like Receptor 2
TNSS	Total Nasal Symptom Score
Treg	Regulatory T Cells
TSLP	Thymic Stromal Lymphopoietin
UAT	Upper Airway Tract
